# Brain functional network changes associated with psychological symptoms in emergency psychological responding professionals after the first wave of COVID-19 pandemic

**DOI:** 10.3389/fpsyt.2023.1014866

**Published:** 2023-04-28

**Authors:** Ying Hu, Hao Hu, Yawen Sun, Yiming Zhang, Yao Wang, Xu Han, Shanshan Su, Kaiming Zhuo, Zhen Wang, Yan Zhou

**Affiliations:** ^1^Department of Radiology, Renji Hospital, School of Medicine, Shanghai Jiao Tong University, Shanghai, China; ^2^Shanghai Mental Health Center, Shanghai Jiao Tong University School of Medicine, Shanghai, China; ^3^Shanghai Key Laboratory of Psychotic Disorders, Shanghai Mental Health Center, Shanghai Jiao Tong University School of Medicine, Shanghai, China; ^4^Institute of Psychological and Behavioral Science, Shanghai Jiao Tong University, Shanghai, China

**Keywords:** COVID-19, emergency psychological responding professionals, psychological symptoms, multiscale functional network, neuroimaging

## Abstract

**Background:**

Emergency psychological responding professionals are recruited to help deal with psychological issues as the Corona Virus Disease 2019 (COVID-19) continues. We aimed to study the neural correlates of psychological states in these emergency psychological responding professionals after exposure to COVID-19 related trauma at baseline and after 1-year self-adjustment.

**Methods:**

Resting-state functional MRI (rs-fMRI) and multiscale network approaches were utilized to evaluate the functional brain activities in emergency psychological professionals after trauma. Temporal (baseline vs. follow-up) and cross-sectional (emergency psychological professionals vs. healthy controls) differences were studied using appropriate *t*-tests. The brain functional network correlates of psychological symptoms were explored.

**Results:**

At either time-point, significant changes in the ventral attention (VEN) and the default mode network (DMN) were associated with psychological symptoms in emergency psychological professionals. In addition, the emergency psychological professionals whose mental states improved after 1 year demonstrated altered intermodular connectivity strength between several modules in the functional network, mainly linking the DMN, VEN, limbic, and frontoparietal control modules.

**Conclusion:**

Brain functional network alterations and their longitudinal changes varied across groups of EPRT with distinctive clinical features. Exposure to emergent trauma does cause psychological professionals to produce DMN and VEN network changes related to psychological symptoms. About 65% of them will gradually adjust mental states, and the network tends to be rebalanced after a year.

## Introduction

In December 2019, an epidemic named COVID-19 occurred in Wuhan, Hubei Province, China (1), and the spread of COVID-19 became a global pandemic, with more than 762 million cases confirmed, and 6.8 million lives lost in over 200 countries as of April 6, 2023.^1^ Compared with the general public, frontline medical staff is exposed to a higher risk of being infected. During the pandemic, medical staff working in the respiratory and emergency department are more likely to suffer anxiety and depression, making them more susceptible to mental disorders (2–6). Besides general medical staff, many psychological professionals volunteered to fight against COVID-19 all through the world (7). To help COVID-19 patients and frontline medical staff cope with their psychological issues, Shanghai Municipal Health Commission has set up the “Shanghai Emergency Psychological Responding Team for supporting Wuhan (EPRT)” to provide psychological assistance. Fifty psychiatrists and psychologists arrived at Wuhan on February 21, 2020, and returned to Shanghai on March 31. Except for the risk of being infected, they also suffer from severe stress when dealing with the traumatic stories of COVID-19 patients and medical staff.

These healthcare workers in emergency care settings are always exposed to stressful environment and trauma, so they are particularly at risk for post-traumatic stress disorder (PTSD) (8, 9). However, controversy exists regarding the exact impact of emergency psychological responses on psychological professionals. Pearlman et al. indicated that psychological therapists who were fledgling or had trauma experience met more psychological difficulties in trauma work (10). Collins et al. found that psychological therapists would manifest symptoms of compassion fatigue burnout, when dealing with their clients’ severe trauma, which influenced their life satisfaction and the quality of psychotherapy (11). Another study demonstrated that psychotherapists showed a high degree of vicarious trauma during the COVID-19 (12). Besides, some researchers believe that treating trauma victims will make psychological professionals more resilient to psychological problems (13). Therefore, the EPRT could be a unique sample for analyzing the impact of emergency psychological responses on psychological professionals because of their very similar background and homologous stress. The comprehensive understanding of the neural activities among EPRT not only helps explore the abnormal fluctuations of these emergency psychological professionals in severe accidents and prevent them from having serious psychological problems, but also helps people better cope with future global health security and improve emergency response capacity of infectious diseases. It will be critical to account for these mental health consequences among for frontline psychiatrists and psychologists facing the current COVID-19 pandemic.

Previous studies have demonstrated that resting-state fMRI (rs-fMRI) could be considered a promising imaging modality to diagnose and evaluate many mental diseases (14–16). Through modern network theory, it is revealed that brain networks constructed from rs-fMRI play an important role in brain function and diseases, and complex nervous systems can be studied from the perspective of brain functional networks. The modular structure is one of the most prominent properties of human brain network, and it balances the separation and integration of brain functions by topologically composing a group of interconnected brain regions (17, 18). Some modular organizations of brain functional networks correspond to well-known functions (19), such as the visual (VIS), somatomotor (SOM), auditory, attention, and default mode systems. There are few studies on how the brain functions of emergency psychological professionals change such as executive, emotional, and memory functions; therefore, further investigations into the alterations in EPRT at the functional modular level may advance our knowledge of the neural mechanism of EPRT.

This work reports the results of a longitudinal project in emergency psychological responding professionals assessed by clinical assessments and rs-fMRI data after supporting Wuhan. The aims were to: (i) investigate functional network alterations at baseline in EPRT; (ii) analyze longitudinal progression trends of brain functional network between EPRT subtypes based on rs-fMRI data; and (iii) explore the impacts of trauma experience and its underpinning neural basis in emergency psychological responding professionals. We hypothesized that alterations of multiscale functional networks in EPRT would be correlated with changes of psychological symptoms, and subsequent improvement of psychological symptoms would be underpinned by the dynamic alterations of brain functional network.

## Materials and methods

### Participants

Our initial sample included 85 participants—46 EPRT members, and 39 healthy controls (HC). All participants were aged between 18 and 55, right-handed, and had an education of at least 15 years. All participants were screened with Mini International Neuropsychiatric Interview (M.I.N.I.) and free of current and past psychiatry diseases. Other exclusion criterions included: substance abuse or dependency within 6 months, contraindication to MRI scan, pregnancy, history of neurological disorders, and family history of mental illness. Three EPRT members was excluded for incomplete MRI or movement artifacts. One subject was identified as outlier of the Posttraumatic Stress Disorder Checklist for DSM-5 (PCL-5) total score and excluded from our present study. Besides, the local psychiatrists from Shanghai were enrolled as HC and matched the EPRT members with age and gender. All HCs were never exposed to COVID19 patients or highly suspected ones. Seven subjects were excluded either for the lack of clinical assessment or the poor image quality. All study procedures were approved by the Institutional Review Board of Shanghai Mental Health Center, which was in accordance with the Declaration of Helsinki. Written informed consents were provided by all the participants.

**Figure 1:**
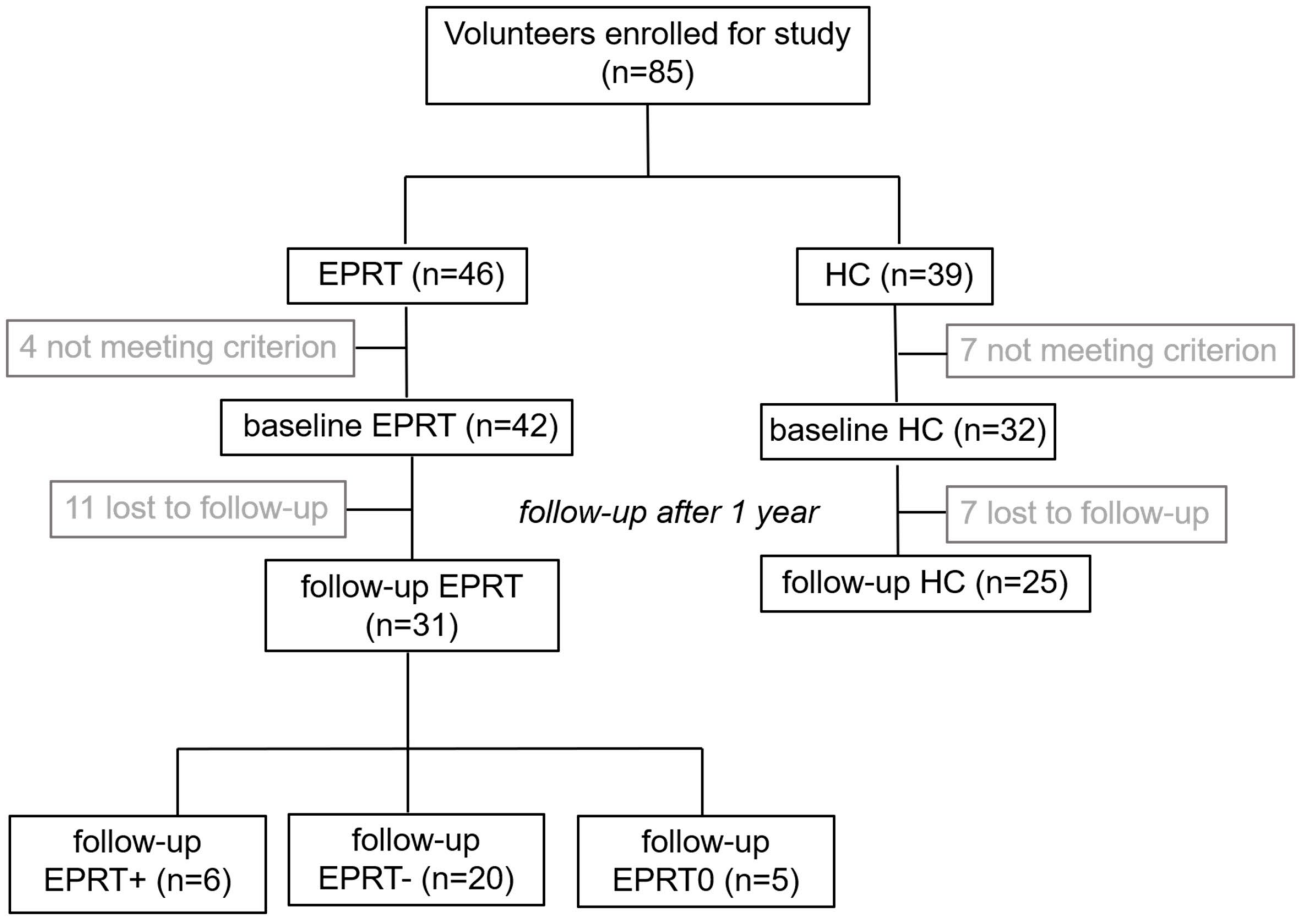
Participant flow through the study.

Due to the loss during follow-up, a longitudinal imaging sample of 31 EPRT members and 25 HCs was included. Among EPRT members, six had an increase in PCL-5 score, 20 had a decrease in PCL, and five had no change in PCL during the 1-year follow-up, so EPRT members were divided into EPRT+ group and EPRT− group according to the changes in PCL-5 scores. Participant flow through the study is presented in Figure 1.

### Clinical assessments

Six psychological scales were applied to assess the clinical features among participants. Patient Health Questionnaire (PHQ9) is a reliable and valid measure of depression severity (20). Generalized Anxiety Disorder 7-item scale (GAD7) is an efficient seven-item self-report scale screening for determining the severity of anxiety in clinical practice and research (21). PCL-5 is a self-report measure to assess the severity of PTSD (22). Interpersonal Reactivity Index (IRI) is a measure of dispositional empathy and had four subscales: perspective taking, personal distress, fantasy, and empathetic concern (23). The Self Reporting Questionnaire (SRQ) is an instrument with 20 simple to understand items which question respondents about symptoms and problems likely to be present in those with neurotic disorder (24). Perceived Stress Scale (PSS10) is widely used for measuring psychological distress (25).

### Imaging data acquisition and preprocessing

MRI data were obtained with 3 T Magnetom Scanner (Siemens, Prisma, United States) at the Radiology Department of Renji Hospital. Eyes opened resting-state fMRI images were acquired using the echo-planar imaging (EPI) sequence. The EPI sequence parameters were listed as follows: acquisition time 452 s, repetition time 2,000 ms, echo time 30 ms, flip angle 90°, voxel size 3.3*3.6*2.4 mm, field of view 230*230 mm^2^, and number of slices 70 and 220 time points. Structural images were acquired with magnetization-prepared rapid acquisition with gradient echo (MPRAGE) sequence, with the following parameters: acquisition time 221 s, repetition time 1,800 ms, echo time 2.28 ms, flip angle 8°, voxel size 1*1*1 mm, field of view 256*256 mm^2^, and number of slices 160.

All rs-fMRI data were preprocessed using DPARSF software (26). The first 10 volumes of each image were removed for longitudinal magnetization stabilization. The remnant images were corrected for slice-timing and realigned the head motions using a six-parameter linear transformation. Then, each functional image was spatially aligned to corresponding T1-weighted MRI image and registered to Montreal Neurological Institute space with a resolution of 3 × 3 × 3 mm^3^. Next, the normalized images were smoothed using a Gaussian filter with 6-mm full-width at half maximum, and its linear trend was removed by temporal linear regression. Also, a set of noise signals were regressed, including the average signals of white matter, average signals of cerebrospinal fluid and the Friston 24-parameter of head motion. At last, rs-fMRI data were bandpass filtered (0.01–0.08 Hz) to reduce physiological artifacts. For each dataset, motion correction was checked to ensure that the maximum absolute shift did not exceed 2 mm, and the maximum absolute rotation did not exceed 2°. There were no significant group differences in head motion (*t* = 1.648, *p* = 0.209). We further evaluated frame-wise displacement (FD), which was defined as the sum of the absolute derivative values of realignment parameters. The maximum FD was <0.5 mm with a mean ± SD across all participants of 0.118 ± 0.09. So, motion scrubbing was not performed to remove the spike volume.

### Functional network construction

**Figure 2:**
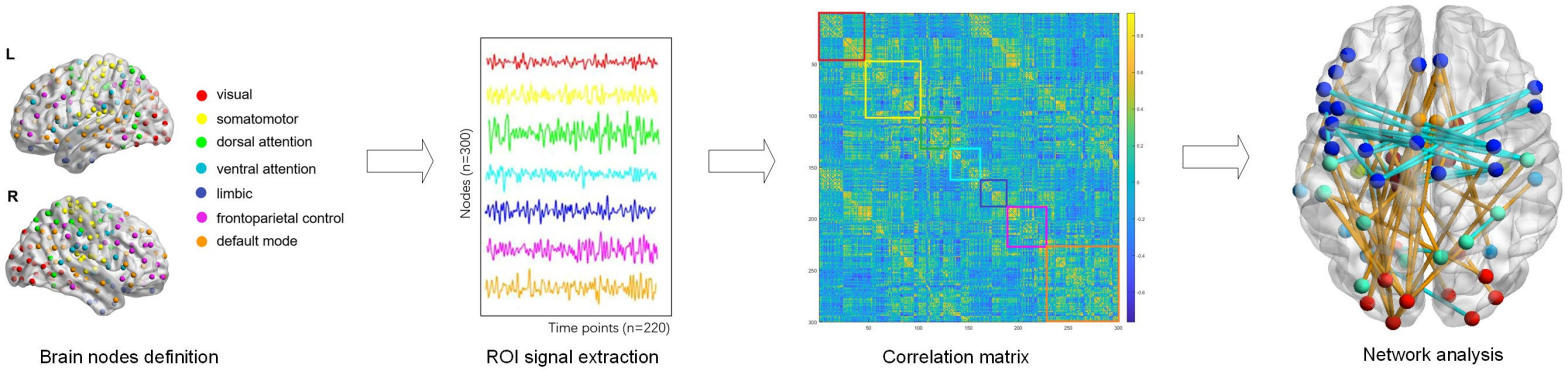
Functional network construction and graph analysis.

The whole cerebral cortex was parcellated into 300 functionally homogenous regions based on the Schaefer-300 template. Each brain region was classified as one of seven network: the VIS, SOM, dorsal attention (DOR), ventral attention (VEN), limbic (LIM), and frontoparietal control modules (FCP), and the default mode network (DMN), according to the canonical seven-module parcellation given by Yeo et al. (27) (Figure 2). The region labels and their matching networks in the seven-module parcellation are shown in Supplementary Table S1.

The brain functional network was constructed by a weighted adjacency matrix where nodes corresponded to brain regions and edges corresponded to functional connectivity between network nodes. Intramodular connectivity represented the connection strength within the module, and the intermodular connectivity between two modules represented the strength connecting both modules. Intra- and inter-modular connectivity provide information about the organization and integration of brain networks, which can help us understand how the brain processes information and supports cognition. Besides, the absolute weighted functional network for each subject was used to calculate the following topological properties: betweenness centrality, degree centrality, clustering coefficient, nodal efficiency, local efficiency, assortativity, hierarchy, local network efficiency, global network efficiency, and small-world. Nodal metrics, which have one value for every region in each subject, include betweenness centrality (BC), degree centrality (DC), clustering coefficient (CC), nodal efficiency (NE), and local efficiency (NLE). Global metrics which have one value for each subject, are assortativity, hierarchy, local network efficiency (Eloc), global network efficiency (Eglob), and small-world properties. In order to investigate the complete connectivity patterns related to psychological symptoms, we calculated the area under curve (AUC) for topological properties using a range of sparsity from 0.10 to 0.40 for each subject. This AUC index for each topological property is calculated to provide a scalar independent of a specific threshold to reduce the influence of different imaging devices and their parameters (28), and is sensitive to topological comparison. All graph measures were calculated using the Graph Theoretical Network Analysis Toolbox (GRETNA) (29).

### Statistical analysis

Continuous variables were analyzed by Student’s *t*-test, and categorical data were analyzed by chi-square test. Since the ages, gender, and years of education were no significant difference in participants, we compared the network properties (the functional connectivity strength and topological properties) between EPRT members and HC at baseline. In addition, because the EPRT members was divided into EPRT+ group and EPRT− group according to the changes in PCL-5 scores during follow-up, the paired *t*-test was used to test specific within-group (EPRT+ and EPRT−) time effects, and the two sample *t*-test was used to test between-group baseline and follow-up differences. In order to further understand the reasons for the changes in psychological symptoms in the EPRT group after 1 year, we also compared the brain functional activities of the EPRT- and non-EPRT groups, as well as the EPRT- and HC groups. We also used paired *t*-tests to compare the relevant measures at baseline and follow-up for HC group. The multiple comparison corrections for *p* values used the false discovery rate (FDR) method (*q* < 0.05), and uncorrected *p* < 0.05 was considered a trend. All statistical analyses were performed by MATLAB R2019b. Additionally, associations between cross-sectional and longitudinal network metrics that showed significant differences and clinical measures were explored using Pearson correlation coefficients. The significant level was set to *p* < 0.05, two-sided.

## Results

### Clinical and demographic measures at baseline

The clinical and demographic features of our recruited sample were illustrated in Table 1. There were no significant differences between EPRT and HC groups in age, gender, and education levels. The total score of PHQ9, GAD7, and IRI did not differ between groups. The total score of PCL-5 in the EPRT group was significantly higher than the score in the HC group (*p* = 0.045). Moreover, substantially more subjects in the EPRT group felt affectively fragile and were stuck in a constant state of sadness, especially exposed to COVID-19 affairs. For the scale of IRI, there were no significant differences between groups.

**Table 1:** Demographics and clinical characteristics of the participants.

	Initial sample (*n* = 74)		Longitudinal subsample (*n* = 60)			
	EPRT	HC	EPRT+	EPRT−	EPRT0	HC
Sample size	42	32	6	20	5	25
Age (SD)	40.98 (6.29)	40.34 (5.82)	41.67 (5.25)	40.8 (6.05)	37 (8.41)	43.36 (5.12)
Gender(M/F)	11/31	8/24	2/4	5/15	1/4	5/20
Education (SD)	16.74 (1.36)	16.94 (1.39)	16 (0)	17.15 (1.53)	16.6 (1.2)	16.84 (1.37)
SRQ (SD)	2.83 (3.50)	1.03 (1.42)	2.67 (2.93)	1.5 (2.54)	0.4 (0.49)	1.2 (1.83)
PHQ9 (SD)	3.48(3.59)	2.13 (2.79)				
GAD7 (SD)	1.74 (2.11)	1.41 (2.04)				
PSS10 (SD)	10.14 (5.56)	7.69 (3.49)				
PCL5 (SD)	6.67 (7.47)	2.80 (3.02)	7.86 (6.54)	3.55 (6.37)	0.8 (1.6)	1.48 (2.71)
IRI total (SD)	34.45 (13.70)	34.38 (10.39)				

SRQ, self-reporting questionnaire; PHQ9, patient health questionnaire; GAD7, generalized anxiety disorder 7-item scale; PSS10, perceived stress scale; PCL-5, posttraumatic stress disorder checklist for DSM-5; and IRI, interpersonal reactivity index.

### Baseline functional connectome in EPRT

#### EPRT vs. HC

**Figure 3:**
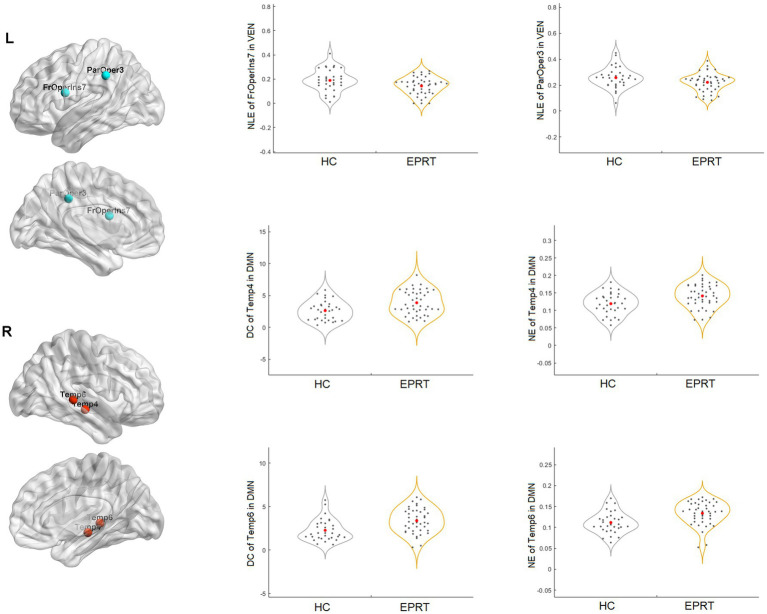
Brain regions with significant differences between EPRT and HC at baseline.

No significant difference in the intramodular and intermodular connectivity strength of the functional network was found between EPRT vs. HC groups. In contrast, graph analysis showed significantly reduced NLE of frontal operculum insula_7 (FrOperIns_7) and parietal operculum_3 (ParOper_3) areas involving VEN and increased DC and NE of temporal_4 (Temp_4) and temporal_6 (Temp_6) areas involving DMN in EPRT groups relative to controls (see Figure 3).

#### EPRT+ vs. EPRT−

No difference was found in global and nodal functional topological properties as well as in intramodular and intermodular connectivity strength between EPRT+ and EPRT− groups at baseline.

#### EPRT− vs. non-EPRT−

Significant difference in the connectivity strength of the DMN and LIM intermodular functional network was found between EPRT− vs. non-EPRT− groups. Graph analysis showed increased DC of temporal pole_2 (TempPole_2) and CC of orbital frontal cortex_1 (OFC_1) area involving LIM in EPRT− group relative to non-EPRT− group.

#### Correlations between baseline functional network alterations and clinical measures

**Figure 4:**
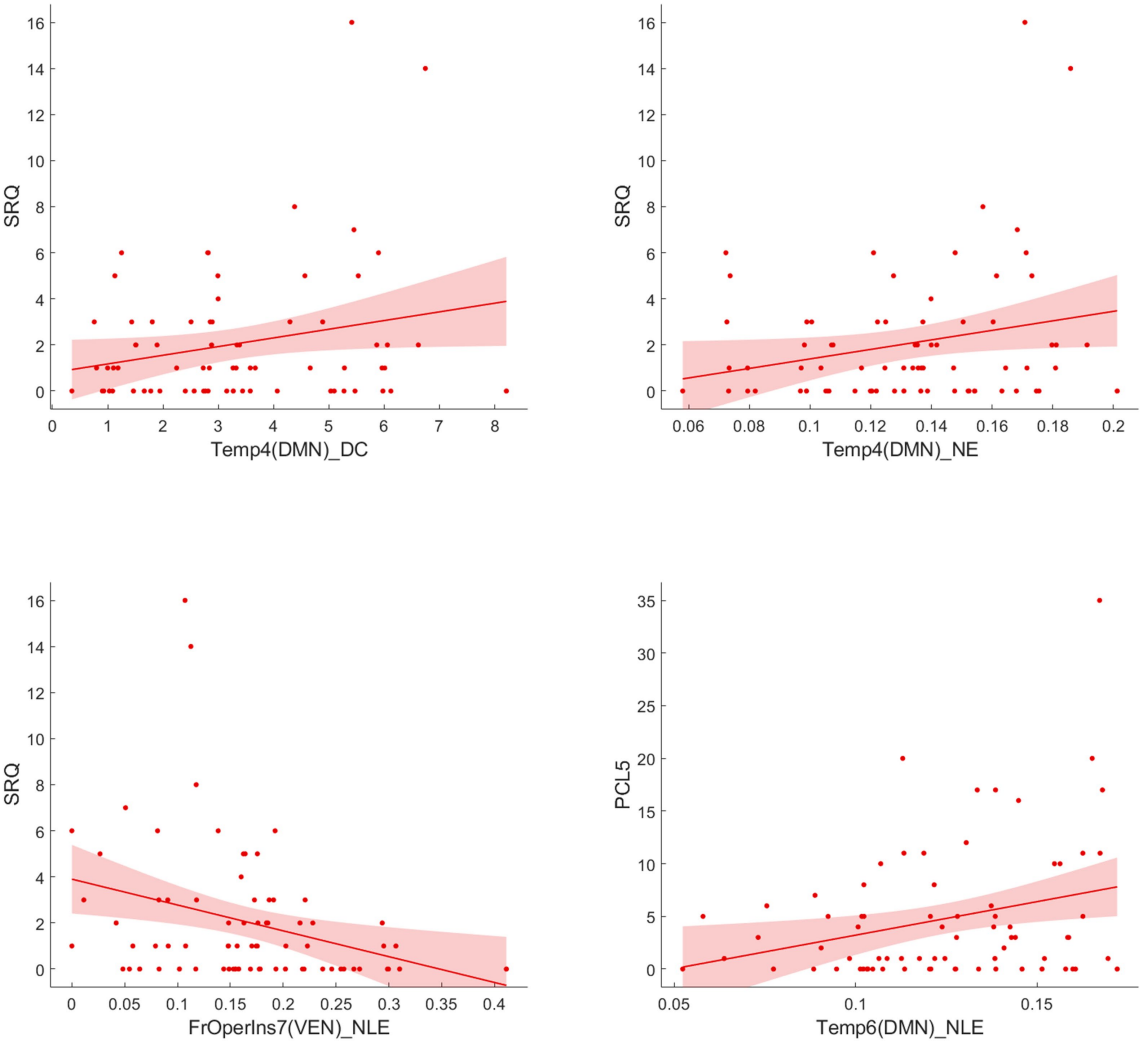
Correlation analysis between significant network metrics and clinical characteristics at baseline.

Following the Pearson correlation analysis, we found that the SRQ was correlated with DC (*r* = 0.2316, *p* = 0.033) and NE (*r* = 0.2330, *p* = 0.0319) of temporal_4 (Temp_4), and PCL-5 was correlated with NE (*r* = 0.2496, *p* = 0.0212) of temporal_6 (Temp_6) involving DMN in participants. Besides, SRQ was inversely correlated with frontal operculum insula_7 (FrOperIns_7; *r* = −0.2711, *p* = 0.012) involving VEN. However, these correlations did not persist after FDR correction. Figure 4 showed the trends between baseline functional network alterations and clinical measures in participants.

### Longitudinal functional connectome changes in EPRT within-group (EPRT+, EPRT− and HC) temporal evolution

In EPRT+ group, no statistically difference was found in global and nodal functional topological properties as well as in intramodular and intermodular connectivity strength over time.

**Figure 5:**
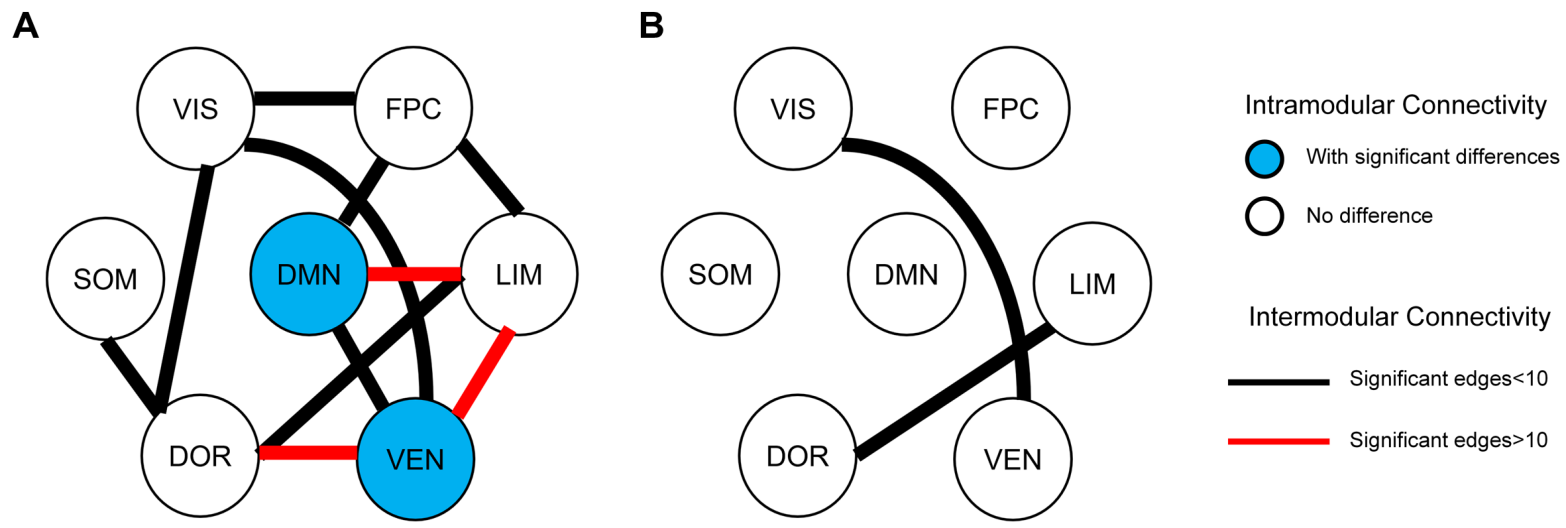
Intramodular and intermodular connectivity changes at follow-up. **(A)** Baseline vs. follow-up among EPRT- group; **(B)** EPRT+ vs. EPRT− at 1 year follow-up.

**Figure 6:**
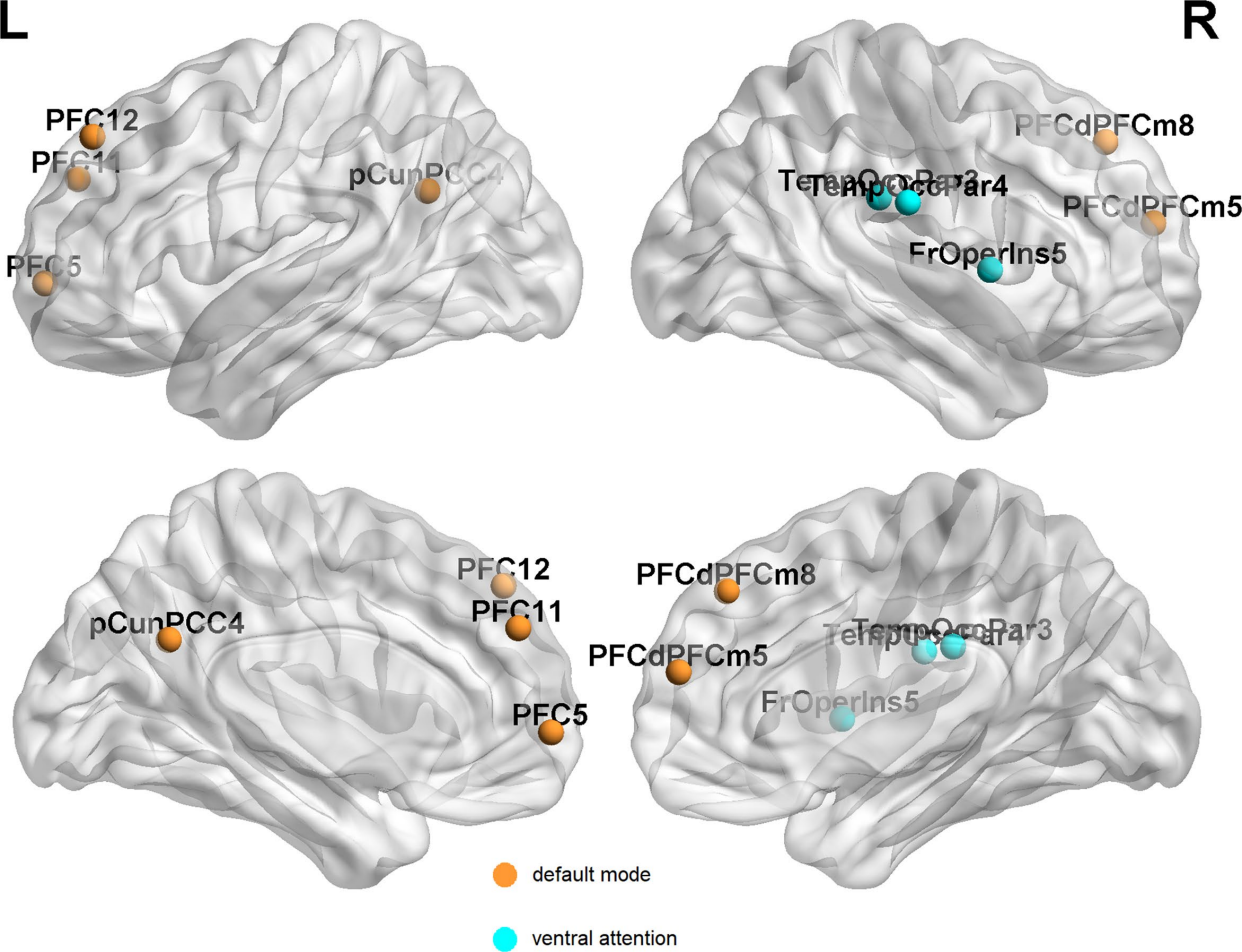
Brain regions with more than one graph metric changes after 1 year follow-up among EPRT- group. PFC, prefrontal cortex; pCunPCC, precuneus posterior cingulate cortex; PFCdPFCm, dorsal prefrontal cortex and medial prefrontal cortex; FrOperIns, frontal operculum insula; and TempOccPar, temporal occipital and temporal parietal.

In EPRT− group, statistically differences were found in intramodular connectivity strength involving DMN and VEN and intermodular connectivity strength over time (see Figure 5A). Additionally, nodal graph properties changed in EPRT− members involved brain areas in DMN, LIM and VEN after 1 year follow-up (see Table 2). Figure 6 illustrated brain regions with more than one graph properties changes over time among EPRT- group.

**Table 2:** Brain regions with graph metric changes after 1 year follow-up among EPRT- group (FDR corrected).

Module	Region	Hemisphere	Nodal graph metrics	*p* value	Baseline > Follow-up
DMN	Temp_1	L	NLE	0.0275	Y
	Temp_6	L	NE	0.0036	Y
	Par_1	L	NE	0.0378	Y
	Par_4	L	NLE	0.0393	Y
	PFC_2	L	NLE	0.0204	Y
	PFC_4	L	NE	0.0424	Y
	PFC_5	L	NE	0.0409	Y
		L	NLE	0.0399	Y
	PFC_11	L	DC	0.0109	Y
		L	NE	0.0036	Y
		L	NLE	0.0275	Y
	PFC_12	L	DC	0.0109	Y
		L	NE	0.0036	Y
		L	NLE	0.0335	Y
	PFC_17	L	NE	0.0394	Y
	pCunPCC_2	L	NE	0.0409	Y
	pCunPCC_4	L	NE	0.0424	Y
		L	NLE	0.0335	Y
	PFCdPFCm_1	R	NLE	0.0204	Y
	PFCdPFCm_5	R	DC	0.0092	Y
		R	NE	0.0036	Y
		R	NLE	0.0275	Y
	PFCdPFCm_7	R	NLE	0.0280	Y
	PFCdPFCm_8	R	NE	0.0381	Y
		R	NLE	0.0086	Y
LIM	TempPole_1	R	DC	0.0233	N
VEN	TempOccPar_3	R	DC	0.0434	Y
		R	NE	0.0354	Y
	TempOccPar_4	R	DC	0.0225	Y
		R	NE	0.0309	Y
	FrOperIns_5	R	DC	0.0225	Y
		R	NE	0.0344	Y
	Med_4	R	DC	0.0434	N

Temp, temporal; Par, parietal; PFC, prefrontal cortex; pCunPCC, precuneus posterior cingulate cortex; PFCdPFCm, dorsal prefrontal cortex and medial prefrontal cortex; TempPole, temporal pole; FrOperIns, frontal operculum insula; and TempOccPar, temporal occipital and temporal parietal.

In HC group, no significant differences between baseline and follow-up measures. This suggests that any changes observed in EPRT group are likely due to the emergency psychological responses rather than normal changes over time.

#### Between-group (EPRT+ vs. EPRT−) follow-up differences

No follow-up difference was found in global and nodal functional topological properties between EPRT+ and EPRT− groups. However, statistically differences were found in intermodular connectivity strength involving VIS-VEN and DOR-LIM between EPRT+ and EPRT− groups at follow-up (see Figure 5B).

#### Between-group (EPRT− vs. HC) follow-up differences

No follow-up difference was found in global and nodal functional topological properties between EPRT− and HC groups.

#### Correlations between longitudinal functional network alterations and clinical measures

**Figure 7:**
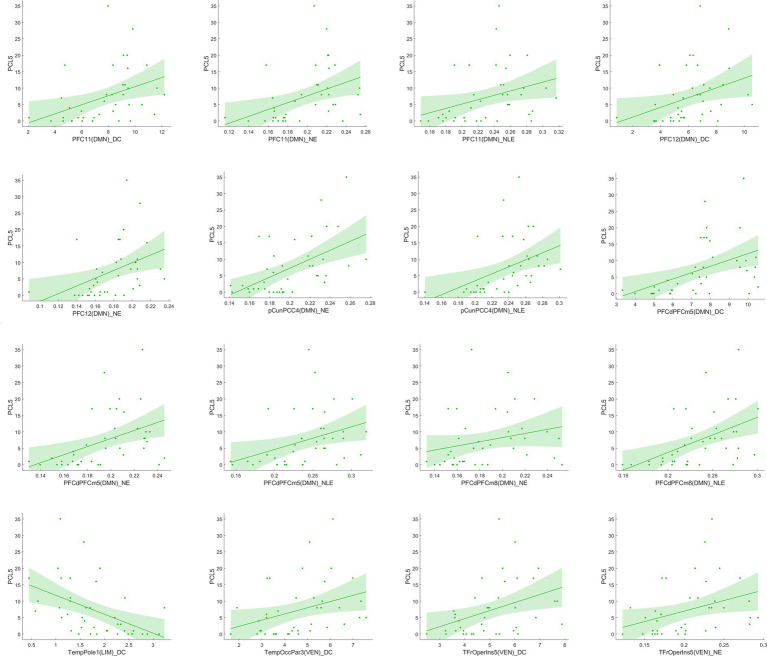
Correlation analysis between significant network metric changes and clinical characteristics after 1 year follow-up among EPRT- group.

Figure 7 reported significant correlations between longitudinal functional network alterations and clinical measures after FDR correction.

## Discussion

To the best of our knowledge this is the first complete longitudinal study to analyze the brain functional network changes in emergency psychological responding professionals exposed to COVID-19 pandemic using graph analysis and connectomics based on rs-fMRI data. The analysis based on module-level network helps facilitate biological interpretation and reduce the complexity of rs-fMRI data compared to analysis based on regional network. Brain functional network alterations and their longitudinal changes varied across groups of EPRT with distinctive clinical features. We investigated that the DMN and VEN module tended to be critical systems both at baseline and 1 year follow-up. A key finding was that network changes were associated with psychological symptoms like stress and depression, anxiety and psychosomatic complaints. Collectively, our findings suggest the impacts of psychological symptoms and its underpinning neural basis in emergency psychological responding professionals exposed to trauma.

In the baseline clinical assessments, the PCL-5 score of the EPRT group was significantly higher than that of the HC group, indicating that the EPRT members were still under stress about 40 days after returning from the epidemic area. They only showed mild stress symptoms, and none of them met the criteria for PTSD. We speculate that these participants are experts in psychology, so they could better regulate their negative emotions and present superior trauma resistance compared with the general public to some extent. More interestingly, we analyzed the brain functional activities between the EPRT− group and the non-EPRT− group, and found that the brain networks related to LIM had discrepancy at baseline. Whether or not the psychological symptoms improvement may be related to the activity of LIM system in people exposed to trauma. This result may indicate that the LIM system is an important loop of resilience ability to adapt and respond to stress or trauma, which is consistent with previous studies (30, 31).

In line with a previous cross-sectional rs-fMRI study (32), we found increased DC and NE of middle temporal gyrus (Temp_4 and Temp_6) areas involving DMN in people exposed to trauma compared to HC at baseline. Middle temporal gyrus is an important region implicated in the pathophysiology of PTSD and has a role in distance contemplation, known face recognition and audio-visual emotional recognition. A previous fMRI study (33) showed increased connectivity in middle temporal gyrus after recent trauma, and another study found functional connectivity between the middle temporal gyrus and amygdala predicted subsequent trauma-related valence (34). Besides, reduced NLE of frontal operculum insula_7 (FrOperIns_7) and parietal operculum_3 (ParOper_3) areas involving VEN. Frontal operculum insula has a role in planning behavior, cognition, and thought, and is associated with primary somatosensory and motor function. The altered graph properties about VEN may be related to VEN’s participation in social cognition, episodic memory (35, 36), and the control of unnoticed or unexpected stimuli to trigger attention shifts (37). The correlation between significant network metrics and clinical characteristics (see Figure 4) indicated that these functional network changes were not only specific to EPRT or HC, but also could represented the psychological symptoms of participants. Until now, few studies have used graph analysis to study functional networks in emergency psychological responding professionals experienced trauma, but our baseline findings are consistent with possible alterations in brain mechanisms following traumatic experience. Among other things, our study has shown an association between higher NE/DC of Middle temporal gyrus, and a higher severity of posttraumatic complaints and stress, may indicate a compensatory mechanism that forms clusters to maintain mental health.

Resting-state fMRI changes were associated with psychological evolution in EPRT, especially in EPRT- group. Paired *t*-test analyses of EPRT- group showed greater psychological symptom reduction was implicated in an increased functional role of the DMN module as an incohesive system (decreased DC, NE, and NLE). This is in keeping with the known role of the DMN module reflecting the neural basis of the self (the autobiographical information, self-emotions) and thinking about others (38), suggesting that what is DMN changed with improvement of psychological symptoms. Lines of evidences suggested that the typical connectivity patterns in DMN module might be altered in clinical populations those with previous significant stress or trauma experience (39, 40). Although there is no one-to-one correspondence between the DMN brain regions involved in the baseline and follow-up results, the changing trend of DMN attributes among EPRT- is consistent with HC’s at baseline and these results may indicate a compensatory rather than a restorative system. Moreover, intermodular connectivity (long connection) changed significantly during the follow-up period in EPRT- group (see Figure 5A), indicating that the functional integration mechanism between the modules may play an important role in the improvement of psychological symptoms. Besides, statistically differences were found in intermodular connectivity strength involving VIS-VEN and DOR-LIM between EPRT+ and EPRT− groups at follow-up (see Figure 5B). It may reflect changes in brain functional network integration between the EPRT- group with improved psychological symptoms and the EPRT+ group with more severe symptoms. Our multiscale network perspective was able to depict trajectories of brain functional changes in line with EPRT stages and progress.

Sripada et al. (41) studied both the DMN and VEN, and found the disequilibrium between these two networks in PTSD. Recent research (42, 43) has proposed that PTSD lead to brain functional alterations involving the VEN, the central executive network, and the DMN. Although EPRT is not sufficient to be diagnosed as PTSD, our findings gave some clues to support this suppose after trauma experience. The results showed that the altered network metrics predominantly overlapped DMN and VEN whether at baseline or during follow-up, which reflects that the topological properties of these two networks were affected or disturbed when psychological symptoms changed. Another interesting finding was that after 1 year follow-up, the EPRT whose psychological symptoms improved (about 65%), the corresponding DMN and VEN gradually tended to rebalance and brain functional activities were not significantly different from HC’s. This indicated that most psychiatrists and psychologists prove to be resilient after being exposed to a traumatic event.

Our study had four limitations. First, we did not collect the clinical data and MRI data before EPRT members’ exposure to COVID-19. The MRI and clinical data were collected 40 days after the EPRT members’ exposure to the COVID-19 patients. Some of their stress-related symptoms probably were relieved by then. Second, intramodular and intermodular connectivity strength results did not survive after FDR correction between baseline EPRT and HC groups, as well as the correlation between baseline functional network alterations and clinical measures. Third, the limited number of time points (220) in the resting state scan is a potential limitation of our study. Finally, our sample is relatively small, especially after 1 year follow-up, which will limit the generalization of the result.

## Conclusion

In conclusion, capitalizing on the comprehensive clinical mental evaluations and the rs-fMRI scans, we identified the impacts of psychological trauma and the brain functional activity alteration in emergency psychological responding professionals. Approximately 65% of EPRT may improve the psychological condition mainly through the significant self-adjustment of DMN and VEN after trauma.

## Data availability statement

The raw data supporting the conclusions of this article will be made available by the authors, without undue reservation.

## Ethics statement

The studies involving human participants were reviewed and approved by Renji Hospital, School of Medicine, Shanghai Jiao Tong University. The patients/participants provided their written informed consent to participate in this study. Written informed consent was obtained from the individual(s) for the publication of any potentially identifiable images or data included in this article.

## Author contributions

YH, ZW, and YZho: study design. HH, YS, and YW: data collection. YH, YS, and HH: analysis and interpretation. YH: drafting of the manuscript. YZha, XH, SS, KZ, ZW, YZho, HH, and YS: critical revision of the manuscript. All authors contributed to the article and approved the submitted version.

## Funding

This research was supported by the National Natural Science Foundation of China (grant no.82171885 and 82001457), Medical Engineering Cross Research Foundation of Shanghai Jiao Tong University (no. YG2022QN037), Shanghai Science and Technology Committee Project (Natural Science Funding: grant no.20ZR1433200, the Explorer Project Funding: grant no.21TS1400700), and Shanghai Rising Stars of Medical Talent Youth Development Program, Youth Medical Talents-Medical Imaging Practitioner Program (grant no. SHWRS(2020)_087).

## Conflict of interest

The authors declare that the research was conducted in the absence of any commercial or financial relationships that could be construed as a potential conflict of interest.

## Publisher’s note

All claims expressed in this article are solely those of the authors and do not necessarily represent those of their affiliated organizations, or those of the publisher, the editors and the reviewers. Any product that may be evaluated in this article, or claim that may be made by its manufacturer, is not guaranteed or endorsed by the publisher.

## Footnotes

^1^ https://covid19.who.int/
